# The question of *the question*: impactful implementation science to address the HIV epidemic

**DOI:** 10.1002/jia2.25898

**Published:** 2022-04-05

**Authors:** Elvin H. Geng, Denis Nash, Nittaya Phanuphak, Kimberly Green, Sunil Solomon, Anna Grimsrud, Annette H. Sohn, Kenneth H. Mayer, Till Bärnighausen, Linda‐Gail Bekker

**Affiliations:** ^1^ Center for Dissemination and Implementation Institute of Public Health Division of Infectious Diseases Department of Medicine School of Medicine at Washington University in St. Louis St. Louis Missouri USA; ^2^ Institute for Implementation Science in Population Health City University of New York New York USA; ^3^ Department of Epidemiology and Biostatistics Graduate School of Public Health City University of New York New York USA; ^4^ Institute of HIV Research and Innovation (IHRI) Bangkok Thailand; ^5^ Primary Health Care, PATH Seattle Washington USA; ^6^ Division of Infectious Diseases Department of Medicine School of Medicine Johns Hopkins University Baltimore Maryland USA; ^7^ International AIDS Society Geneva Switzerland; ^8^ TREATAsia and amfAR Bangkok Thailand; ^9^ The Fenway Institute and Harvard Medical School Boston Massachusetts USA; ^10^ Heidelberg Institute of Global Health Faculty of Medicine and University Hospital Heidelberg Germany; ^11^ Institute of Infectious Disease and Molecular Medicine Desmond Tutu HIV Centre University of Cape Town Cape Town South Africa

**Keywords:** effectiveness research, HIV, implementation science, implementation strategies, public health, research questions

## Abstract

**Introduction:**

Questions about the implementation of evidence‐based intervention to treat and prevent HIV have risen to the top of the field's scientific priorities. Despite the availability of highly efficacious treatment and prevention interventions, impact has fallen short of targets because these interventions are used with insufficient reach, consistency, sustainability and equity in diverse real‐world settings. At present, substantial excitement for implementation science — defined as research methods and strategies to improve use of evidence‐based interventions — has focused on developing and disseminating methods to conduct rigorous research. Yet, impactful answers depend on a sometimes less visible, but even more important, step: asking good questions about implementation.

**Discussion:**

In this commentary, we offer several considerations for researchers formulating implementation research questions based on several distinctive features of the field. First, as findings are used not only by other researchers but by implementers, scientific questions must incorporate a range of stakeholder and community perspectives to be most relevant. Second, real‐world settings are contextually diverse, and the most relevant scientific questions must position answers to make sense within these contexts (whether geographical, organizational and sociological), rather than apart from them. Third, implementation is complex and dynamic; consequently, research questions must make use of emerging standards in describing implementation strategies and their effects whenever possible. Finally, the field of implementation science continues to evolve, so framing problems with a diverse disciplinary lens will enable researchers to pose insightful and impactful questions.

**Conclusions:**

We are now at a juncture marked by both rich evidence‐based interventions and a persistent global pandemic. To achieve continued scientific progress against the HIV epidemic, asking the right questions might be part of the answer itself.

## 1 INTRODUCTION

To be impactful, findings from implementation science to address the HIV pandemic must not only be rigorous, like any scientific claim, but also applicable to and relevant for public health and healthcare practice, which is distinctive. Criteria for rigour are the topic of much discussion – investigators should apply reproducible, vetted and transparent methods to generate credible and objective findings in implementation science through clearly conceptualizing and specifying implementation strategies, use when appropriate of implementation science frameworks and theories, and alignment with reporting standards (e.g. the Standards for Reporting Implementation Studies [StaRI] Statement) [1]. The relevance of specific findings to the settings where they are implemented, however, depends on a different set of issues, which has received less attention to date: the research question itself. Even though every study begins with a question, not all questions yield relevant answers for implementation. We propose several considerations for investigators who seek to use science to advance implementation of evidence‐based interventions in the HIV response.

## 2 DISCUSSION

### 2.1 Part I: Whose perspectives?

The immediate consumers of implementation science are not only other scientists, but also policy makers, implementers, practitioners and communities. Making use of their perspectives in the question‐generating process can help ensure that the answers matter to those affected by the findings.

#### 2.1.1 Start with the end in mind (no matter where you start)

Scientific findings to improve implementation exist at the beginning as well as throughout the translational science spectrum. We need not wait for the regulatory approval or market arrival of novel medications, devices or practices to ask questions about how to use them [2]. For example, one potential approach to HIV cure seeks to render the virus dormant but not eliminated (i.e. so‐called “block and lock” [3]). A hypothetical cure using this approach may require long‐term, and perhaps indefinite, monitoring [4]. Assessing the desirability of such a “cure” to people living with HIV — in particular as compared to today's highly effective and well‐tolerated medications — could inform the prioritization of this direction of inquiry [5]. In another example, novel long‐acting injectable formulations of antiretroviral therapy (ART) are generating much excitement [6]. But the story of long‐acting injectable or implantable antipsychotics offers a cautionary tale. Also widely heralded, injectable antipsychotics fell short of anticipated impact due to both the inability of outpatient clinics to deliver injections (i.e. limiting supply) and patient fears that users would be labelled as non‐adherent or “bad” patients (i.e. limiting demand) [7]. Studying how to influence provider and patient acceptance can pave the way for uptake even before the medications become available [8]. Starting (i.e. during discovery and development) with the end in mind (i.e. delivery and desirability) can accelerate impact.

#### 2.1.2 Incorporate a range of end‐user voices into formulating scientific questions

A number of methods in implementation research help bring end‐users into formulation of research questions. People living with HIV have led the scientific response to HIV in both practical (e.g. accelerating scientific review at the U.S. National Institutes of Health) and conceptual ways (e.g. driving scientific priorities) [9]. Human‐centred design is a method that explicitly brings designers and end‐users together, and starts with fostering empathy and ideation as a prerequisite for successful co‐creation [10]. Human‐centred design is applied to both refine questions about implementation and seek solutions [11]. Crowd sourcing — enlisting input from large numbers of people from the public or a community — represents a novel method now with demonstrated effectiveness in the design of HIV services (e.g. HIV testing in China) [12]. Healthcare workers are another group of end‐users who are critical for informing questions about the complex integration of innovations into complex process. Methods such as Intervention Mapping can specify how to convene a multi‐stakeholder process, and can be deployed to bring diverse perspectives to bear on formulating a question about implementation [13]. In short, end‐user engagement through a range of participatory methods can enhance the impact of implementation research in HIV.

#### 2.1.3 Interrogate systems

Implementation gaps often result from friction between systems and people. Research questions to close these gaps can problematize individual behaviour (e.g. “poor patient adherence” or “bad provider decisions”). Yet, excess demands on people often result from inadequate, burdensome and inefficient systems. In the absence of a critique of systems, implementation science may mistakenly problematize the behaviour of individuals to compensate for faulty systems. When patients are not retained in HIV care, some may see the problem as one of inadequate patient education or insufficient motivation. Instead, the challenge could be re‐conceived as being due to service‐delivery systems that are not accessible nor high‐quality enough to engage patients. When front‐line healthcare workers underperform, questions could examine provider motivation or skills. Alternatively, research questions could investigate negative workplace culture, restricted autonomy and environmental stressors [14]. Awareness of systems can help to ensure that our questions do not implicitly and inadvertently locate the problem and, therefore, the burden of improvement unfairly on individuals within those systems.

#### 2.1.4 Question not only failures of implementation but implementation of failure by design

Implementation science would be remiss if it did not expose systems designed to discriminate, mis‐implement and impede. Paul B. Batalden, a leader in the field of quality improvement, observed that “Every system is perfectly designed to get the results it gets [15].” In that light, today's implementation gaps can also be viewed through the lens of discriminatory systems that underpin pervasive disparities. For example, in the United States, Black Americans have higher prevalence of HIV and greater mortality as compared to White Americans, but their use of both ART for treatment and pre‐exposure prophylaxis for prevention is lower [16]. Implementation science can be used to reveal these structures, including through analyses that name racism as a driver of differential access to healthcare [17]. In some states or countries, criminalization of HIV transmission or same‐sex relations themselves beget discriminatory practices [18]. Research about service delivery may also reflect uninterrogated standards that normalize inequity. For example, cost‐effectiveness research may peg “cost‐effectiveness” to per capita income, which implicitly accepts an assumption that those who are poor are consigned to less. A research agenda intended to advance health of populations must also uncover systems that disadvantage the same people [19] they are intended to support.

### 2.2 Part II: What questions are being asked?

Questions in implementation research are most useful when they focus on *implementation*. While this argument seems axiomatic, much research in the HIV field still gives preference to clinical interventions and outcomes. Research that makes implementation strategies clear and reports implementation outcomes — and thereby bring implementation itself into clear scientific focus — is urgently needed.

#### 2.2.1 Go beyond effectiveness studies to ask about the implementation strategies themselves

Even though “effectiveness” studies carried out in “real‐world” populations are more representative of potential population‐level effects than “efficacy” research, such studies do not optimally inform practice when they do not ask questions about *implementation strategies* used to change systems in order to better deliver evidence‐based interventions. When studies assign activities to implement a novel intervention to research staff using protocolized activities [20], they yield limited knowledge on how to implement such activities in real‐world organizations or systems after the study is over. Emerging standards in implementation science encourage investigators to specify who is carrying out a strategy (i.e. the health systems actor), the activities in that strategy (i.e. actions) along with their dose and timing, the health system target (i.e. action target) and other dimensions help to ensure implementation itself is the object of study [21]. Effectiveness studies can show that an intervention “works” in a more representative population, but questions about the implementation activities themselves will inform how to actually implement in practice.

#### 2.2.2 Study de‐implementation of low‐value or harmful beliefs and practices

Given that most service delivery settings are often already operating at full capacity, research to improve implementation must also identify inefficient or ineffective practices to eliminate. For example, early global HIV programs funded by donors often included abstinence programs, which research showed to be ineffective, but nevertheless lingered for years because of donor belief systems [22]. In another example, while early studies in 2000 found that 95% adherence was needed for treatment success, this figure was based on regimens, including first‐generation, upboosted protease inhibitors that are no longer used [23]. Some of today's medications may require drug levels consistent with 60–80% adherence for suppression [24, 25]. Yet, the position that HIV treatment success requires 95% adherence and that lower levels of adherence justify withholding treatment remains present in some settings [26]. Studying how to de‐implement practices based on outdated data can help optimize the impact of the whole system [27].

#### 2.2.3 Ask questions about context to make findings about implementation strategies more relevant to diverse implementing settings

Context is often said to be king in implementation. If so, explicit questions about the context (i.e. setting‐specific features that affect *the effects* of implementation strategies) should accompany implementation research. For example, consider a trial that finds that audit‐and‐feedback successfully accelerates provider initiation of ART. By investigating contextual features influencing success, such a study might also find that the effects of audit and feedback are attenuated in clinics where providers have limited confidence about performance data because of weak data storage and management systems. Such an observation would imply that potential adopters should assess provider confidence in information systems before investing in audit‐and‐feedback as an improvement strategy. A study that finds the same overall effects of audit‐and‐feedback, but which does not ask about the impact of credibility of performance data, will be unable to inform potential adopters of this key contextual factor, which could result in poor reproducibility. In a research study, questions to identify contextual drivers of success will help implementers identify promising practice settings for adoption.

### 2.3 Part III: How are questions asked?

Even though implementation research values context, the research community should also avoid the “context trap” in which we conceive of every setting as irreducibly unique. To strike the right balance, we can make full use of emerging scientific perspectives that facilitate sharing insights across settings.

#### 2.3.1 Use shared terminology and measurements about implementation whenever possible to facilitate progress

One of the major contributions of implementation science is putting forth the concept of *implementation outcomes* that represent the effects of all implementation strategies, no matter how different from one another they are [28]. Such shared implementation outcomes include acceptability, appropriateness, penetration and sustainability, and others enable shared research questions across specific implementation strategies. Instruments to quantify implementation outcomes, such as acceptability [29] and sustainability [30], now exist and can be applied to different implementation strategies to bring out shared findings. To illustrate, consider two strategies: one to enhance paediatric engagement in HIV care through instituting a “family clinic day,” where services are oriented towards whole‐family care [31], while a second seeks to improve HIV testing through secondary distribution of HIV self‐tests by pregnant women to male partners [32]. While a dedicated clinic day and secondary test distribution are fundamentally different activities, if both can be examined through common implementation outcomes (e.g. acceptability to patients, providers and communities; sustainability), shared insights about what may be possible and accelerate science to make progress against the HIV pandemic.

#### 2.3.2 Seek generalizable insights even where a single generalizable effect is not plausible

Clinical researchers often — and justifiably — assume that clinical interventions have meaningful “average” effects that apply across organizational settings. For example, a meta‐analysis of randomized trials showed that use of glucocorticoids reduced the risk of death by 50% in moderate‐to‐severe AIDS‐related *Pneumocystis jirovecii* pneumonia. Such findings are considered widely applicable irrespective of hospital system, organizational culture, geography or even patient socio‐demographic characteristics (e.g. age, sex and race) [33]. On the other hand, we cannot assume that implementation strategies have invariant effects across settings, organizations and patient populations. This absence of a single effect does not, however, imply that generalization is impossible, but does mean research must seek and identify factors that enable generalizing. For example, in the START‐ART stepped‐wedge trial [34], a multi‐level implementation strategy to accelerate ART initiation demonstrated the heterogeneity of effects across the 20 health centres involved. Accompanying qualitative research found that the strategy was most effective in facilities where formal healthcare workers had strong working relationships with peer health workers, who in turn spread the expectation of rapid ART initiation in the community, suggesting that effects are likely maintained in environments where these peer cadres are strong, but attenuated where peer providers are absent. Understanding the conditions and mechanisms that enabled the strategy to work allows qualified and bounded, but nevertheless potentially wide‐ranging, generalization.

#### 2.3.3 Draw from a broad range of methodologies for questions about implementation even if they are not branded as implementation research

Calls to use recognizable methods from implementation science should not preclude the HIV field from using a broader set of methods that could also help address implementation problems. The social sciences (e.g. economics, psychology and sociology) offer rich insights into human and organizational behaviour that form the foundations for today's implementation research theories and frameworks [35, 36]. For example, Rogers’ diffusion of innovations theory emerged from the field of sociology and contributes to the Consolidated Framework for Implementation Research. Pawson and Tilley's Realistic Evaluation approach focused on context–mechanism interactions and foreshadowed today's interest in understanding the mechanisms of implementation strategies [37]. Weick's studies of “dropping” unneeded tools in organization psychology anticipated today's conversation about de‐implementation [38]. Scholarship from social sciences has contributed to empirically tested approaches to enhance the uptake of evidence‐based interventions, such as use of pastors as opinion leaders to encourage the use of HIV prevention interventions, use of incentives to increase HIV testing [39, 40] and harnessing what Cialdini identified as the psychological reflex of reciprocity [41] (the cognitive impulse to return a favour) for public health through pay‐it‐forward schemes [40] for sexually transmitted infection testing. Diverse scientific insights can advance implementation even if not named as implementation science.

## 3 CONCLUSIONS

The remarkable scientific successes of HIV clinical research have created the possibility of widespread impacts on population health. However, to make the most of this opportunity for impact requires a re‐focusing of scientific priorities on questions that address implementation. Impactful questions require end‐user input and stakeholder engagement, must be interpretable in specific implementing contexts, seek mechanistic insights and bounded generalizability, and draw from diverse disciplines. To make continued progress against the HIV epidemic through impactful research, it turns out that asking the right questions might be a big part of the answer itself.

## COMPETING INTERESTS

EHG: Educational grant from ViiV Healthcare.

LGB: Honoraria for an advisory role to Merck PTY LTD, Gilead Sciences and ViiV Healthcare.

AHS: Research and community grants from ViiV Healthcare.

KHM has received unrestricted research grants from Gilead Sciences and Merck, Inc, and has served on scientific advisory boards for Gilead Sciences, Merck, Inc and ViiV Healthcare.

## AUTHOR CONTRIBUTIONS

EHG and LGB conceived of the premise and drafted the initial manuscript. KG, NP, DN, SS, AG, TB, AHS and KHM contributed to critical review and revision of the paper. All authors have read and approved the final manuscript.

## FUNDING

None.

## DISCLAIMER

None.

## Data Availability

No empiric data here so not applicable.
